# 

**Published:** 1912-04

**Authors:** 


					﻿Vol. XXVII
APRIL, 1912

TEXAS
medic^^B
JOURNAL
Established
July, 1885
“Red Back”
PUBLISHED MONTHLY AT AUSTIN, TEXAS, BY
F. E. DANIEL, M. D., - - - Editor ancb^OpHtitw*
PRINTED BY THE VON BOECKMANN-JONES CO.
Subscription, 81.00 a Year, in Advance. -	-	-	-
Entered at the Postoffice at Austin, Texas, as second class mail matter.
				

